# The basis for self-reported sexual dysfunction and the diagnostic value of masturbation-related parameters in men who have not engaged in vaginal intercourse in the past 6 months or have never engaged in it: a comparative study

**DOI:** 10.1093/sexmed/qfaf087

**Published:** 2025-10-26

**Authors:** Zhihui Mao, Juanhui Ye, Karl H Pang, Chunlin Wang, Yan Zhang

**Affiliations:** Department of Infertility and Sexual Medicine, The Third Affiliated Hospital of Sun Yat-sen University, Guangzhou 510630, Guangdong, China; Ruikang Clinical Medical College, Guangxi University of Chinese Medicine, Nanning 530001, Guangxi, China; Division of Surgery and Interventional Science, University College London, Gower Street, London WC1E 6BT, United Kingdom; Endocrinology & Medical sexology (ENDOSEX) Department of systems Medicine, University of Romer Tor Vergata, 00133 Rome, Italy; Department of Infertility and Sexual Medicine, The Third Affiliated Hospital of Sun Yat-sen University, Guangzhou 510630, Guangdong, China

**Keywords:** sexual dysfunction, vaginal intercourse, masturbation, Masturbation Erection Index (MEI), Masturbatory Premature Ejaculation Diagnostic Tool (MPEDT)

## Abstract

**Background:**

Men who have not engaged in vaginal intercourse in the past 6 months or have never engaged in it often seek help for sexual dysfunction, identifying factors influencing patients’ self-assessment of sexual function and the value of masturbation-related parameters in diagnosing sexual dysfunction is of great importance.

**Aim:**

This study aims to understand the reason why patients self-report sexual dysfunction and evaluate the role of masturbation parameters in diagnosing sexual dysfunction in self-reported sexual dysfunction (SRSD) and self-reported no sexual dysfunction (SRNSD) groups.

**Methods:**

Our study was conducted mainly by filling out a questionnaire, which collected demographic information, sexual history as well as sexual parameters. The questionnaire summarized the basis of patients' self-reported sexual dysfunction and also included the Erection Hardness Score (EHS), Masturbation Erection Index (MEI), Masturbatory Premature Ejaculation Diagnostic Tool (MPEDT), and masturbatory ejaculation latency time (MELT).

**Outcomes:**

The main outcomes were reasons for SRSD individuals to judge their sexual dysfunction, the EHS, MEI, MPEDT, and MELT scores.

**Results:**

The most common complaints included insufficient erection hardness and short ejaculation latency time during masturbation, with 84.85% of self-reported erectile dysfunction and 91.80% of self-reported premature ejaculation patients reporting these issues. No significant difference was found between past vaginal sexual experiences (6 months ago) and current self-reported sexual dysfunction. Significant differences were found in EHS, MEI, MPEDT, and MELT scores between the SRSD and SRNSD groups. The MEI showed a sensitivity of 89.29% and a specificity of 81.82%. The MPEDT demonstrated a sensitivity of 98.04% and a specificity of 72.73%.

**Clinical Implications:**

We proposed that other than vaginal intercourse, sexual dysfunction should also be assessed from noncoital sex and verified the scientific validity of the masturbation parameters in people without recent vaginal intercourse.

**Strengths & Limitations:**

We firstly explored the patients self-perceived basis for sexual dysfunction. However, the objective instruments were not employed in diagnosing sexual dysfunction.

**Conclusion:**

The findings emphasize the importance of a comprehensive clinical assessment that includes evaluating masturbation, noncoital sex (between men and women), morning erections, and past vaginal sexual experiences (6 months ago), moreover, masturbatory scales provide valuable insights in diagnosing sexual dysfunction.

## Introduction

Male sexual dysfunction encompasses a spectrum of disorders that affect one or more stages of the male sexual response cycle, including male hypoactive sexual desire disorder, erectile disorder, premature ejaculation (PE), delayed ejaculation, among others.^1^ These conditions can significantly impact an individual's quality of life and may cause personal distress for the sexual partner.^2^^,^^3^ In clinical practice, it is not uncommon for men who have never had or have not engaged in vaginal intercourse for at least 6 months to seek help for sexual dysfunction. In male sexual dysfunction, the most prevalent complaints are erectile dysfunction (ED) and PE.^4^^,^^5^ The general definitions of ED and PE are based on the premise of vaginal intercourse, therefore, these definitions are not applicable to men with no experience of vaginal intercourse.^6^^,^^7^

The common diagnostic tools for ED and PE are also not suitable for these patients. Assessment scales such as the International Index of Erectile Function (IIEF-5) and the Premature Ejaculation Diagnostic Tool (PEDT) have been widely used worldwide.^8-10^ However, the questions on these scales are based on vaginal intercourse within the past 6 months. Therefore, if a patient has not engaged in vaginal intercourse in the past 6 months, it is inappropriate to use these scales. Understanding how patients self-assess their sexual dysfunction and scientifically evaluating their current sexual function are both critical, yet challenging.

In the broader scope of sexual health, sexual activities include, but are not limited to, penetrative vaginal intercourse, as well as noncoital activities such as kissing, touching, frottage, masturbation, and oral sex.^11-13^ According to previous findings, masturbation is a common sexual practice worldwide,^14-16^ and it is more evident among individuals who are solo. Other sexual activities, such as oral or anal sex, are also prevalent.^13^ A survey found that 89.8% of people have had oral sex recently and 19.0% have had a history of anal sex on heterosexual population.^17^ However, at present, there are no adequate assessment parameters for oral sex, and the population is minor in anal sex. Therefore, assessing sexual function using masturbation parameters may address the majority of sexual activity complaints and provide a unified criterion for individuals who have never had vaginal intercourse or have not engaged in it in the past 6 months.

For patients reporting ED, the Erection Hardness Score (EHS) and the Masturbation Erection Index (MEI) are appropriate tools to assess erectile function.^18^^,^^19^ The EHS can be used to screen for organic ED during masturbation and has been shown to have a close relationship with successful sexual intercourse.^20^^,^^21^Additionally, morning erections, being the final phase of nocturnal penile tumescence, are considered a reflection of erectile function and are commonly believed to signify good sexual health.^22-24^ The MEI is a scale derived from the IIEF-EF and the EHS, specifically used to evaluate erectile function during masturbation, and it has been proved to have a high internal consistency.^19^ For patients reporting PE, the masturbatory ejaculation latency time (MELT) and the Masturbatory Premature Ejaculation Diagnostic Tool (MPEDT) are valuable parameters, although the concept still needs to be more widely accepted globally.^25^^,^^26^ The ﻿Italian Society of Andrology and Sexual Medicine (SIAMS) released an innovative official guideline, advocating the use of MELT as part of the clinical assessment for diagnosing PE patients.^27^ The MPEDT, derived from the PEDT, has been validated as an objective diagnostic tool for evaluating PE during self-masturbation.^25^ Although these tools show great efficacy in distinguishing between individuals with sexual dysfunction and those without, they are not yet widely used in clinical settings.

## Methods

### Participants and study design

All the participants were recruited from December 20, 2023, to April 2, 2024. Participants with self-reported sexual dysfunction (SRSD) were recruited in the Department of Infertility and Sexual Medicine at the Third Affiliated Hospital of Sun Yat-sen University, Guangzhou, China. Self-reported no sexual dysfunction (SRNSD) participants were recruited from the general population through a public online platform. Inclusion criteria for participants were: (1) Men aged 18 to 60 years; (2) Who have never had, or have not had vaginal intercourse in the past 6 months; (3) SRNSD or SRSD (including self-reported PE or ED); (4) Heterosexuality. Exclusion criteria for participants were: (1) Other sexual disorders besides PE or ED (such as decreased libido, delayed ejaculation); (2) Significant psychiatric diseases; (3) The use of drugs affecting sexual function in the past 6 months (including antidepressants, androgens and testosterone preparations). (4) Reproductive organ malformations affecting sexual function (including penile curvature, hypospadias). All participants provided informed consent and were informed that they could withdraw from the study at any time. Our study was approved by the Ethics Committee of the Third Affiliated Hospital of Sun Yat-sen University (trial registration number: II2023-271-02).

In our andrology clinic, individuals with SRSD were initially subjected to a comprehensive medical history assessment, with particular emphasis on their sexual history, as this is a crucial factor in diagnosing sexual dysfunction, especially PE, which is largely diagnosed based on sexual history.^6^^,^^7^^,^^28^^,^^29^ For individuals with SRNSD, potential participants were randomly invited offline from healthy volunteers. Those who agreed to participate were provided with a link to the online survey platform. The questionnaire included a range of essential data, such as demographic information (age, BMI, educational status), sexual history, and sexual parameters. It took about 20 minutes to finish the questionnaire. 82 SRSD and 44 SRNSD participants were invited to fill the questionnaires. Finally, we recruited 112 effective questionnaires, 71 with SRSD and 41 with SRNSD.

### Statistical analysis

All the data were analyzed using IBM SPSS Statistics, version 27.0 (IBM Corp., Armonk, N.Y., USA). Qualitative data were presented as absolute and percentage frequencies. Quantitative data were presented as the mean ± standard deviation (SD) or medians (interquartile range, 25th and 75th percentiles) for normally and non-normally distributed variables, respectively. Normal or non-normal distribution was determined using the Shapiro–Wilk test. Independent Student's t-test or Mann–Whitney U-test were used to compare differences in normally or skewed distributed data, respectively. The Chi-square test was used to assess the correlation between sexual history (6 months ago) and current self-reported sexual status. Differences were considered statistically significant at *P* < 0.05.

## Results

### Demographic data

Demographic data are presented in Table 1. Regarding educational status, all participants had at least a junior high school diploma, with the majority holding a bachelor's degree, followed by junior college degree, and the fewest holding a PhD. The 71 SRSD population [26.00 (22.00, 28.00)] (years) was slightly older than the 41 SRNSD population [25.00 (24.00, 26.50)] (years) (*P* = 0.403). The BMI was significantly higher in the SRNSD group (*P* = 0.026).

**Table 1:** Demographic characteristics.

	SRSD (n = 71)	SRNSD (n = 41)	All	*p*
**Age** (year)	26.00(22.00, 28.00)	25.00(24.00, 26.50)	25.00(22.00, 28.00)	0.403
**BMI**(kg/m^2^)	21.37(19.38, 24.16)	22.41(21.30, 24.52)	22.07(20.12, 24.42)	0.026
**Educational status**				<0.001
Primary school	0(0.00%)	0(0.00%)	0(0.00%)	
Junior high school diploma	9(12.68%)	0(0.00%)	9(8.04%)	
Senior school or technical secondary school	9(12.68%)	1(2.44%)	10(8.93%)	
Junior college	23(32.39%)	11(26.83%)	34(30.36%)	
Bachelor’s degree	28(39.44%)	16(39.02%)	44(39.29%)	
Master’s degree	2(2.82%)	12(29.27%)	14(12.50%)	
PhD	0(0.00%)	1(2.44%)	1(0.89%)	
Total	71(100%)	41(100%)	112(100%)	

Values in age and BMI were in Median (P25, P75); Values in educational status was in N(%). Abbreviations: BMI, body mass index; SRSD, self-reported sexual dysfunction; SRNSD, self-reported no sexual dysfunction.

### Masturbation history

**Figure 1:**
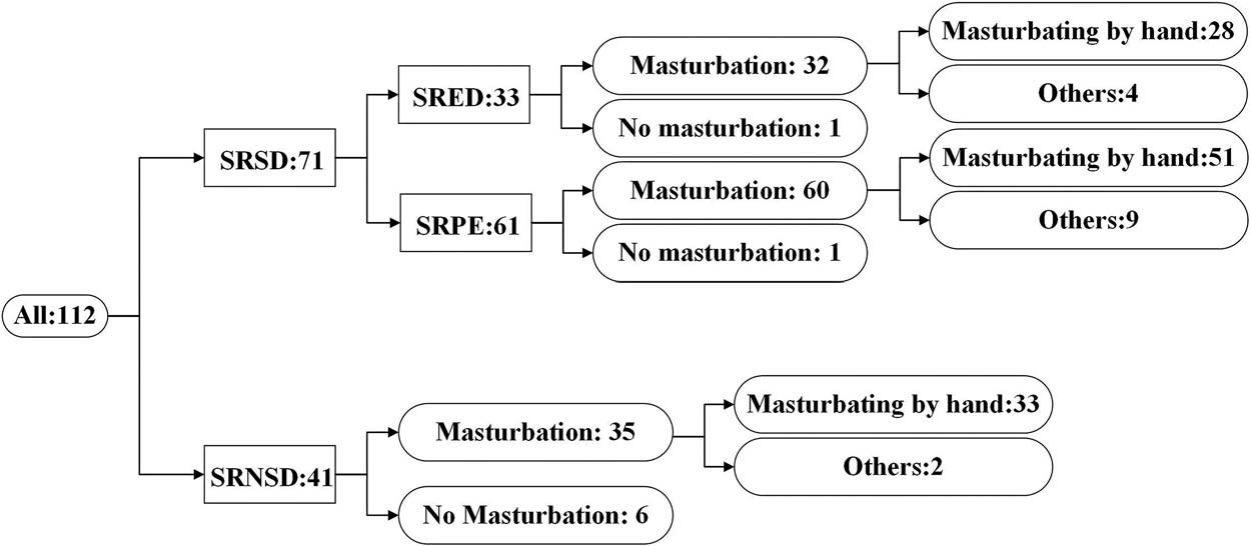
Classification of the participants and the ways of masturbation. Abbreviations: SRSD, self-reported sexual dysfunction; SRNSD, self-reported no sexual dysfunction; SRED, self-reported erectile dysfunction; SRPE, self-reported premature ejaculation.

Of the two groups, 69 men (97.18%) in the SRSD group and 35 men (85.37%) in the SRNSD group reported a history of masturbation (Figure 1). The median (IQR) frequency of masturbation in the SRSD and SRNSD groups was [6.00 (4.00, 10.00)] times per month vs [7.00 (4.00, 10.00)] times per month (*p* = 0.441). The most common method of masturbation was stroking or rubbing the penis by hand, also called typical masturbation. A minority of participants also reported engaging in atypical forms of masturbation, such as masturbating by squeezing legs, or practicing prone masturbation. Only a few participants reported using a masturbator or sex toy.

The SRSD group (N = 71) consisted of three subgroups: 10 people with self-reported erectile dysfunction (SRED) only, 38 people with self-reported premature ejaculation (SRPE) only, and 23 with both SRED and SRPE. For further analysis, we consolidated the three subgroups into two main groups: the SRED group (n = 33) and the SRPE group (n = 61). In the three groups, 32 in the SRED group masturbated, 60 in the SRPE group masturbated, and 35 in the SRNSD group masturbated. 28 people in the SRED group adopted typical masturbation, accounting for 87.50% of the masturbating population. In the SRPE group, 51 people adopted typical masturbation, accounting for 85.00% of the masturbating population. In the SRNSD group, 33 people used typical masturbation, accounting for 94.29% of the masturbating population.

### The common reasons for SRSD

**Figure 2:**
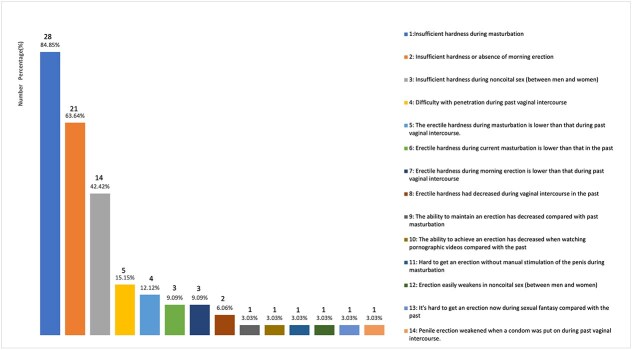
Reasons for self-reported erectile dysfunction (SRED).

**Figure 3:**
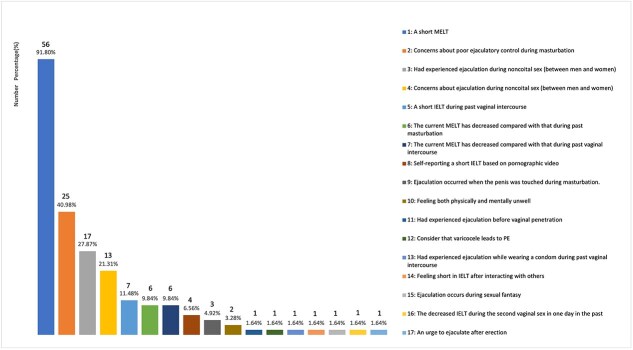
Reasons for self-reported premature ejaculation (SRPE). Abbreviations: MELT, masturbatory ejaculation latency time; IELT, intravaginal ejaculatory latency time; PE, premature ejaculation.

The SRSD population sought medical attention for various reasons (Figures 2 and 3). The reasons for reporting sexual dysfunction were generally categorized into five groups: masturbation, noncoital sex (between men and women), past vaginal sexual experiences (6 months ago), morning erection, and others (Table 2). Problems with masturbation were the most common reason reported by both groups, accounting for 90.91% in the SRED group and ﻿91.80% in the SRPE group. Regarding noncoital sex (between men and women), 42.42% of the SRED group reported issues with erectile hardness, while 32.79% of the SRPE group reported ejaculatory problems. 21.21% and 14.75% reported problems in past vaginal sexual experiences (6 months ago) in the SRED and SRPE groups. Additionally, 66.67% of the reasons reported by the SRED group were related to morning erections.

**Table 2:** General classification of reasons for SRSD patients.

	**SRED (ranks)**	**N (%)**	**SRPE (ranks)**	**N (%)**
Masturbation	1,5,6,9,11	30(90.91%)	1,2,6,7,9	56(91.80 %)
Noncoital sex (between men and women)	3,12	14(42.42%)	3,4	20 (32.79%)
Past intravaginal sexual experience	4,8,14	7(21.21%)	5,11,13,16	9(14.75%)
Morning erection	2,7	22(66.67%)	-	-
Others	10,13	2(6.06%)	8,10,12,14,15,17	9(14.75%)
Total population		33(100.00%)		61(100.00%)

Values were in N (%). Ranks: see it in Figures 2 and 3. **Abbreviations:** SRSD, self-reported sexual dysfunction; SRED, self-reported erectile dysfunction; SRPE, self-reported premature ejaculation.

The reasons for the SRSD individuals to report their sexual dysfunction varied between the two groups. For the SRED population (Figure 2), 84.85% reported insufficient hardness during masturbation. Additionally, 63.64% reported insufficient hardness or absence of morning erections, followed by 42.42% who reported insufficient hardness during noncoital sex (between men and women).

In the SRPE population (Figure 3), the most frequently reported reason for seeking medical treatment was a short MELT, which was reported by 56 men (91.80%). Additionally, 25 individuals (40.98%) expressed concerns about poor ejaculatory control during masturbation. Furthermore, 27.87% had experienced ejaculation during noncoital sex (between men and women), while 21.31% expressed concerns about ejaculation during noncoital sex (between men and women).

### Other uncommon reasons for SRSD

There were a few less common but notable reasons. The SRED population included: compared with the past, the ability to achieve an erection has decreased when watching pornographic videos (3.03%) and compared with the past, it's hard to get an erection now during sexual fantasy (3.03%).

The SRPE population included: self-reporting a short intravaginal ejaculatory latency time (IELT) based on pornographic video (6.56%), feeling both physically and mentally unwell (3.28%), consider that varicocele leads to PE (1.64%), feeling short in IELT after interacting with others (1.64%), ejaculation occurs during sexual fantasy (1.64%), an urge to ejaculate after erection (1.64%).

### Past sexual experience

Both the SRED and SRPE populations reported problems in the past sexual experiences (21.21% and 14.75%, respectively, for general classification) (Table 2). Among the SRSD population, 37 individuals (52.11%) reported no vaginal intercourse in the past. In comparison, 26 participants (63.41%) from the SRNSD group also disclosed a lack of such experience. The Chi-square test showed no correlation was found between sexual history (6 months ago) and current self-assessed sexual function (Table 3).

**Table 3:** Crosstab of the self-reported sexual function status and the past sexual experiences, Chi-square test showed *P* > 0.05.

	**Never had intravaginal sexual intercourse, n (%)**	**Had intravaginal sexual intercourse in the past (more than** 6 **months ago), n (%)**		*p =* 0.245
SRSD	37(52.11%)	34(47.89%)	71(100%)	
SRNSD	26(63.41%)	15(36.59%)	41(100%)	
	63	49	112	

**Abbreviations:** SRSD, self-reported sexual dysfunction; SRNSD, self-reported no sexual dysfunction.

### Masturbation parameters

To objectively compare masturbation parameters between the SRSD and SRNSD groups, we only included people with typical masturbation. Between the SRED and SRNSD groups, the MEI and the EHS were statistically significant (Table 4). Regarding the MEI, 25 individuals in the SRED group scored below the established cutoff, indicating a sensitivity of 89.29%. Conversely, 27 individuals in the SRNSD group scored above the cutoff, reflecting a specificity of 81.82%. Additionally, the EHS for masturbation in the SRNSD group was significantly higher than that of the SRSD group. Besides, 26 (78.79%) experienced morning erections in the SRED group and 35 (85.37%) in the SRNSD group. The EHS of morning erections in the SRNSD group was also significantly higher than that of the SRED group. Among the SRPE subjects, 50 men were diagnosed with PE by the MPEDT, demonstrating a high sensitivity of 98.04%. Additionally, 24 SRNSD individuals were identified as normal by the MPEDT, yielding a specificity of 72.73%. These findings indicated that the MELT and the MPEDT scores were significantly different between the SRPE and SRNSD groups (Table 4).

**Table 4:** The masturbation parameters of the participants in each group.

	**SRPE (n = 51)**	**SRNSD (n = 33)**	*p*
MELT/min	2.50 (1.00, 3.00)	10.00 (7.00, 19.50)	<0.001
MPEDT	15.00(12.00, 17.00)	5.00 (4.00, 6.00)	<0.001
	**SRED (n = 28)**	**SRNSD (n = 33)**	
MEI	19.50 (14.00,23.75)	29.00 (28.00, 30.00)	<0.001
EHS (masturbation)	2.00 (2.00,3.00)	4.00 (3.50,4.00)	<0.001
	**SRED (n = 26)**	**SRNSD (n = 35)**	
EHS (morning erection)	3.00 (2.00,3.00)	3.00 (3.00,4.00)	=0.001

Values were in Median (P25, P75). **Abbreviations:** MELT, masturbatory ejaculation latency time; MPEDT, Masturbation Premature Ejaculation Diagnostic Tool; MEI, Masturbation Erection Index; EHS, Erection Hardness Score.

## Discussion

In our study, participants provided valuable insights into the diagnosis of sexual dysfunction in the SRSD group. The findings revealed the multifaceted factors influencing individuals' decision to seek medical intervention for sexual dysfunction. The statistical results showed that there were significant differences in masturbation parameters between the SRSD and SRNSD groups. Meanwhile, the diagnostic scales showed high sensitivity.

For the demographic data, the educational level result showed all the subjects were junior high school or above, which ensured that the subjects could adequately understand the content of the questionnaire. The BMI was higher in the SRNSD group. Although a large number of studies showed that obesity was positively associated with a range of sexual health issues, however, Hasan Karadag et al. (2014) demonstrated that after controlling for the presence of cardiovascular disorders, diabetes, hypertension, thyroid diseases, anxiety and depression, obesity was not associated with any sexual dysfunction in male population, moreover, the medians of BMI in both groups were in healthy weight range(18.5–24.9)﻿kg/m^2^.^30^ In this case, the difference in BMI between the two groups can be considered negligible.

The motivations for seeking consultation in the SRSD group highlighted specific concerns about their sexual function. The SRED and SRPE patients both regarded masturbation as the primary basis for judging sexual dysfunction, accounting for 90.91% and 91.80% respectively (Table 2). This indicates that masturbation is the main way for the SRSD patients to assess their sexual function. Among the SRED group, the hardness during masturbation is an important reference indicator, accounting for 84.85%. Similarly, previous study used masturbation hardness for the initial screening of organic ED, which further demonstrated the importance of masturbation hardness in the assessment of erectile function.^21^ Among the SRPE population, a striking 91.80% of respondents reported a short ejaculation latency time during masturbation, followed by concerns about poor ejaculation control, coupled with the fact that the patients were disturbed by the unsatisfactory performance of masturbation. These findings closely aligns with the ISSM definition of PE: a short ejaculation latency time, loss of control, and personal distress.^6^ To some extent, masturbation plays a significant role in assessing PE, suggesting that the diagnostic criteria for PE could also be applicable to the act of masturbation.

Noncoital sex (between men and women) is a common aspect of human sexual activity, encompassing various forms of foreplay. Y.N. Guo et al. (2004) identified six specific types of foreplay: caressing the body, kissing, genital stroking, cuddling, breast stroking, and talking while listening to music or watching TV.^31^ Julián Monge-Nájera et al. (2017) classified noncoital activities as foreplay,^32^ however, their classification did not fully align with Guo et al.'s, as it encompassed a broader range of behaviors. Foreplay is typically considered a prelude to vaginal intercourse, while noncoital sex can occur independently. Stuart Brody et al. (2009) concluded that the frequency of vaginal intercourse, rather than other sexual activities, is more closely associated with sexual satisfaction.^11^ In our study, many participants believed that their performance during noncoital sex could indicate sexual function, with some expressing concerns about preoccupation with ejaculation. Such introspective concerns may affect patients' psychological well-being, potentially leading to negative impacts on male sexual function. This, in turn, could influence their future engagement in vaginal intercourse. These findings highlighted the importance of integrating assessments of noncoital sex and patients' psychological profiles into the diagnostic and therapeutic approach for sexual dysfunction.

Garcia demonstrated that past sexual experiences can influence an individual's choice of a partner for dating and marriage.^33^ However, no studies have definitively demonstrated the impact of past sexual experiences on current sexual function. Our results suggested that an individual's vaginal sexual history does not directly influence their perception of sexual function. For SRSD patients, the impact of past vaginal intercourse experiences over 6 months should be considered less significant. This may differ from conventional understanding, where past vaginal intercourse is often a key factor in sexual history taking. However, it’s easy to neglect the time span, which leads to the deviation of the medical history taking. The mainstream sexual function assessment tools also validated this point, the time span of the IIEF-5 and the PEDT is 6 months, and that of the IIEF-EF is four weeks, all of them do not exceed half a year. As such, their current (no more than 6 months) sexual function and status should be prioritized.

Interestingly, in the SRED population, beyond vaginal intercourse, penile erection in other contexts may also be interpreted as a sign of 'ED.' The top three reasons prompting patients to visit the hospital were insufficient hardness during masturbation (84.85%), morning erection issues (63.64%), and erectile problems during noncoital sex (42.42%). Although the diagnosis of ED based on erectile dysfunction other than vaginal intercourse is not consistent with traditional ED diagnostic criteria, the presence of subclinical symptoms should not be ignored, as this may have a serious impact on the patient's sexual confidence, which could, in turn, affect their sexual confidence in future vaginal encounters. These findings underscore the importance of incorporating the erectile function of masturbation, morning erections and noncoital sex into medical history taking.

The phenomenon of decreased ejaculation latency time during the second vaginal intercourse in one day is another indicator of sexual function. 53.3% of patients with PE engaged in multiple sexual intercourse sessions within a single day to compensate for their short IELT during the first encounter.^34^ Zhang et al. demonstrated that PE patients who attempted vaginal intercourse multiple times in one day reported a significantly increased IELT, and suggested that the diagnostic criteria for PE should be updated.^35^ From the physiological perspective, patients who have multiple intercourse within a day should theoretically have a prolonged IELT during their second sexual intercourse, as multiple sexual activities within a short period of time may lead to an increase in the sexual arousal threshold and longer refractory period. The decreased IELT during the second vaginal intercourse may also indicate diminished sexual function.

There were also some cognitive biases that led to the judgment of sexual dysfunction in the other categories. Self-reported PE or ED through pornographic videos underscores the potential impact of unrealistic media-driven expectations on perceptions of sexual performance. Studies have shown that men who prefer masturbation with pornography are at an increased risk of sexual dysfunction.^36^ Bőthe et al. found that problematic pornography use was linked to sexual functioning issues.^37^ Interestingly, some patients with varicocele believed that it can lead to PE, which in turn raised concerns about PE. Although studies have proved that there are more people with PE among patients with varicocele, there is no direct causal relationship between them.^38^ In addition, some patients self-reported experiencing PE after interacting with others. Others reported sexual dysfunction based on their performance during sexual fantasy. These bases may be deficient in science or even wrong, but they provide an important direction for us to conduct psychological treatment for patients, so as to solve their sexual function problems more effectively.

Our study utilized the most representative masturbation parameters, which demonstrated strong efficacy. Given that atypical masturbation techniques can affect sexual function,^39^^,^^40^ we excluded participants who used atypical methods to eliminate bias when analyzing ejaculation latency time and erectile hardness. Similarly, the participants using masturbation devices were also not included. Higher MPEDT scores and lower MELT in the SRPE population objectively reflected their concerns about PE. Similarly, the MEI between the SRED and SRNSD groups revealed measurable differences in erectile function, with higher EHS scores for the SRNSD population in both morning erection and masturbation, indicating the inferior erectile function of the SRED group. The high sensitivity of the MPEDT and the MEI suggested that these instruments offered a reliable measure for assessing sexual dysfunction.

Our study provides novel insights into why individuals report sexual dysfunction despite not having recent vaginal intercourse. Furthermore, we applied masturbation parameters to assess sexual function in men without recent vaginal intercourse and confirmed their scientific validity. These parameters may serve as convenient and reliable tools for screening and monitoring sexual function in the male population. However, there were some limitations in our study. Firstly, we did not employ objective measures such as RigiScan or electrophysiological testing to evaluate sexual function, and thus, the assessment of the sexual function status of individuals was rather subjective. Additionally, although web-collected data were convenient and efficient, it weakened the accuracy of the researcher's answers to subjective questions, which may lead to a selection bias. For patients with SRSD, it remains unclear whether sexual function assessment based on masturbation parameters aligns with their self-reported results during future vaginal intercourse, and further follow-up is needed. To validate our findings, future studies with larger sample sizes and a multi-center design are warranted.

## Conclusion

Our research demonstrated that, in addition to vaginal intercourse as a direct means of evaluation, patients may use noncoital sex measures to self-assess their sexual function. Furthermore, we found that previous vaginal sexual experiences (6 months ago) had no significant impact on the current sexual function assessment. Actually, the current sexual function deserves more of our attention. For these individuals, using masturbation parameters to evaluate sexual function offers a valuable alternative.
